# A Case Report on Aplasia Cutis Congenita: Insights Into the Impact of Maternal Carbimazole Use

**DOI:** 10.7759/cureus.68663

**Published:** 2024-09-04

**Authors:** Hajar Elmoqaddem, Anass Ayyad, Sahar Messaoudi, Rim Amrani

**Affiliations:** 1 Mother and Child Health Laboratory, Faculty of Medicine and Pharmacy, Mohammed First University, Oujda, MAR; 2 Department of Neonatology and Neonatal Resuscitation, Mohammed VI University Hospital, Oujda, MAR; 3 Department of Neonatology and Neonatal Resuscitation, Faculty of Medicine and Pharmacy, Mohammed First University, Oujda, MAR; 4 Mother and Child Health Laboratory, Faculty of Medicine and Pharmacy, Mohammed First University, Oujda, Oujda, MAR

**Keywords:** abdominal defect, aesthetic, rare anomaly, neonatology, carbimazole, skin grafting, congenital aplasia cutis (cac)

## Abstract

Congenital aplasia cutis (CAC) is a rare neonatal condition characterized by the absence of skin at birth, often associated with diverse underlying conditions. We report the case of a newborn male admitted on the second day of life with a skin defect on the anterior abdominal wall and a lesion on the left thigh. The mother was treated with carbimazole for hyperthyroidism. Notably, there were no similar cases in the family history. The patient showed favorable progress and normal development following a successful dermo-epidermal allograft. Particular attention was given to managing the risk of infection and ensuring optimal healing through tailored wound care protocols. This case underscores the complexity of CAC, highlighting the importance of early diagnosis, multidisciplinary care, and ongoing research to understand better and effectively treat this rare condition.

## Introduction

Congenital aplasia cutis (CAC) is a rare congenital condition, affecting approximately one to three out of 10,000 live births [1], characterized by the absence of skin at birth. While commonly affecting the scalp, CAC can also involve other regions, including the trunk and abdominal wall [1,2]. The etiology of CAC remains unclear, though it is thought to result from a combination of genetic predisposition, vascular disruptions, and teratogenic exposures during pregnancy [2,3]. The management of CAC is complex due to the high risk of complications such as infections and poor wound healing [1].

In this report, we present a case of a newborn male with a skin defect on the anterior abdominal wall and left thigh, whose mother was treated with carbimazole for hyperthyroidism. This case highlights the potential impact of maternal medication exposure, specifically carbimazole, on the development of CAC. It emphasizes the need for a multidisciplinary approach and further research to better understand the pathophysiology and optimal management of this rare condition.

## Case presentation

The patient, a male newborn, was admitted to the neonatal intensive care unit on the second day of life due to a significant skin defect on the anterior abdominal wall. He was born at term following a pregnancy that initially involved twins; however, one twin was lost during the second trimester. Despite this complication, the pregnancy culminated in a normal vaginal delivery. The newborn had normal Apgar scores at birth.

The mother, a 36-year-old multiparous woman, was treated for hyperthyroidism with carbimazole (20 mg/day), which was started at the beginning of the pregnancy and continued throughout its duration. She had no history of hypertension or diabetes, and there was no family history of similar conditions or skin diseases. However, we do not have data on the circumstances of the carbimazole prescription for the mother or the follow-up during the pregnancy.

**Figure 1:**
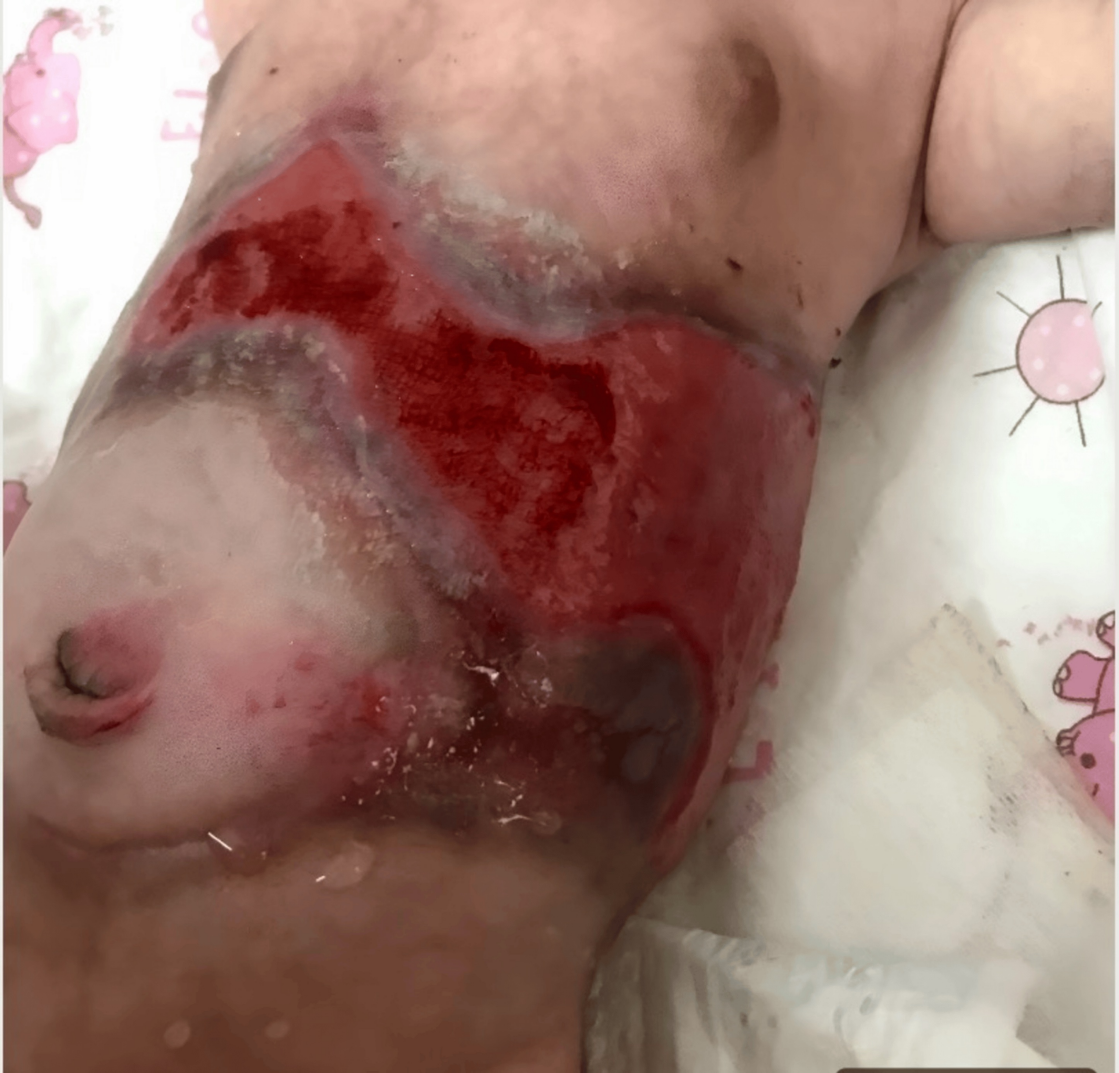
Erythematous hemorrhagic lesion with violaceous borders on the supraumbilical abdominal wall in the newborn patient

**Figure 2:**
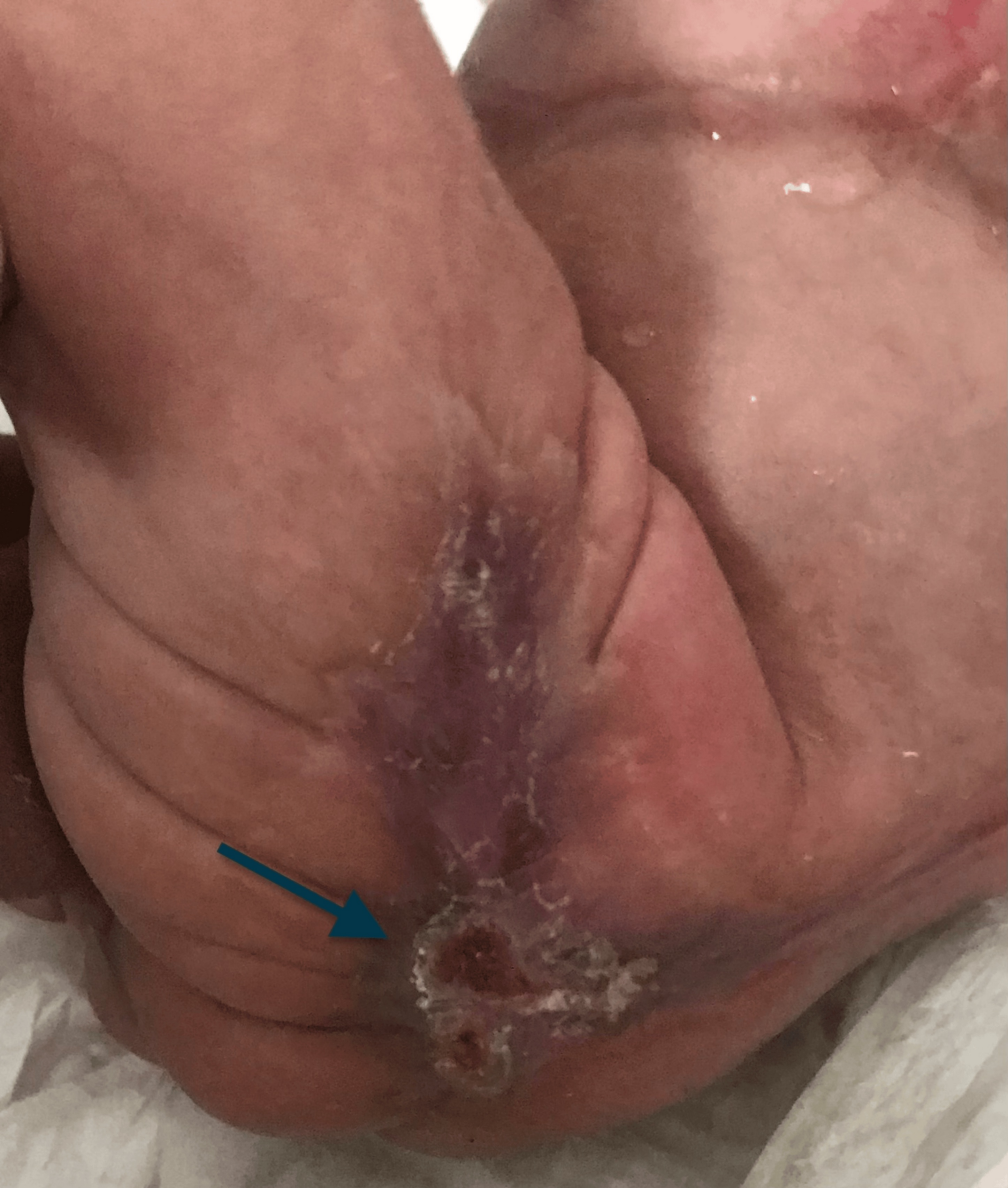
Crusted lesion on the upper left thigh in the newborn patient

 At birth, the newborn had normal anthropometric measurements: a weight of 3,200 grams, a length of 49 cm, and a head circumference of 34 cm, as well as stable vital signs. Physical examination revealed an erythematous hemorrhagic lesion with violaceous borders on the supraumbilical abdominal wall, measuring 15 cm in length. Additionally, a well-demarcated crusted lesion was observed on the upper part of the left thigh (Figures 1, 2). The scalp and mucous membranes were unaffected, and the systemic examination was unremarkable.

Several laboratory investigations, including a complete blood count, electrolytes, liver, and renal function tests, all returned within normal limits (Table 1). Serologic tests for infection were negative (Table 1). Further imaging studies, including abdominal and cranial ultrasound screening and echocardiography, also revealed normal findings.

**Table 1:** Patient's laboratory test results and serological evaluations on admission AST: Aspartate Aminotransferase; ALT: Alanine Aminotransferase; BUN: Blood Urea Nitrogen; TORCH: Toxoplasmosis, Other infections (Syphilis, Varicella-zoster, and parvovirus B19), Rubella, Cytomegalovirus (CMV), and Herpes simplex virus (HSV)

Tests	Result	Reference Range
Complete Blood Count		
White Blood Cell Count	13,450 /µL	10,000 - 25,000 /µL
Hemoglobin	13 g/dL	13.5 - 18.0 g/dL
Hematocrit	37%	35.0 - 60.0%
Platelet Count	214,000 /µL	150,000 - 450,000 /µL
Electrolytes		
Sodium	140 mmol/L	135 - 145 mmol/L
Potassium	4.5 mmol/L	3.5 - 5.0 mmol/L
Liver Function Tests		
ALT	20 U/L	7 - 56 U/L
AST	35 U/L	10 - 40 U/L
Renal Function Tests		
Creatinine	4 mg/L	2.4 - 8.5 mg/L
BUN	20 mg/dL	6 - 36 mg/dL
Additional Serologic Tests		
C-reactive protein (CRP)	8 mg/l	0 - 10 mg/L
TORCH Screening	Negative	Negative
Blood Culture	No growth	No growth
Procalcitonin	0.16 ng/ml	0 - 0.5 ng/mL

Local care with petrolatum gauze was administered until a dermo-epidermal allograft was performed on the 17th day of life. Postoperative recovery was uneventful, and after a three-year follow-up, the patient showed favorable progress and normal development.

Regarding the mother, a specialist consultation was sought after the pregnancy, and the carbimazole treatment was discontinued. No alternative medication was prescribed.

## Discussion

ACC is a rare congenital disorder characterized by the localized or extensive absence of the epidermis, dermis, and occasionally subcutaneous tissue, typically observed at birth [1,2]. The etiology of ACC is complex and involves a combination of genetic, environmental, and teratogenic factors [2-4]. While ACC most commonly presents as an isolated defect, it can also occur alongside other physical anomalies or malformation syndromes. Frieden's classification system categorizes ACC into nine groups based on the location of the lesions and the presence or absence of associated malformations [2].

ACC induced by teratogenic factors, particularly medications, is classified under Frieden's type vIIIa [2]. Among synthetic antithyroid drugs, carbimazole and its active metabolite, methimazole, are the most frequently implicated [5]. The association between ACC and these medications was first identified by Milham and Elledge in 1972 [6]. The risk of congenital anomalies, including ACC, increases when these drugs are administered during the critical period of organogenesis in the first trimester [5]. ACC is not the only malformation associated with antithyroid drugs; other anomalies such as persistent urachus, anal atresia, and choanal atresia have also been reported [5,7].

The causal relationship between ACC and maternal use of methimazole/carbimazole during pregnancy remains debated. Although definitive evidence is lacking, epidemiological and biological plausibility supports the association [5,8]. Additionally, a genetic predisposition to ACC and other embryopathies related to methimazole/carbimazole exposure has been suggested [9].

Regarding the choice of antithyroid drugs during pregnancy, guidelines have evolved in recent years. While propylthiouracil (PTU) has been associated with an increased risk of acute hepatitis [10], it remains the preferred option over carbimazole. However, the risks associated with untreated or inadequately treated hyperthyroidism in pregnant women are more severe. Both the American Thyroid Association and the Endocrine Society recommend PTU as the first-line treatment during the first trimester [11], although this medication is unfortunately not available in Morocco.

The prognosis for ACC can be severe, with mortality rates ranging from 20% to 55% due to serious complications [3]. Secondary infections of the lesions significantly worsen the condition and impact outcomes [1]. Management depends on lesion size and any underlying defects. De Wilde et al. emphasize the importance of early diagnosis and intervention to prevent complications like infections and scarring [12]. While local treatment is often sufficient for small lesions, larger lesions generally require surgical intervention, such as skin grafting or flap procedures [1].

Effective management typically involves a multidisciplinary approach [1], as observed in our case.

## Conclusions

ACC presents significant diagnostic and management challenges due to its rare and multifactorial nature. This case highlights the interplay of maternal teratogenic exposure in the development of ACC, emphasizing the need for a nuanced understanding of this factor. The successful treatment with a combination of conservative and surgical approaches underscores the importance of a tailored, multidisciplinary strategy in managing severe cases.

Following the pregnancy, a specialist consultation was conducted for the mother, resulting in the discontinuation of carbimazole treatment. No alternative medication was prescribed. Regular follow-up was established, and any future pregnancies, if desired, would have to be carefully planned and monitored. This comprehensive approach reflects the broader need for ongoing research and increased awareness, which are crucial for advancing our knowledge and improving outcomes for patients with ACC.
